# Oral involvement in a case of AA amyloidosis: a case report

**DOI:** 10.1186/1752-1947-4-200

**Published:** 2010-06-30

**Authors:** M İnanç Cengiz, Hom-Lay Wang, Levent Yıldız

**Affiliations:** 1 Zonguldak Karaelmas University, Faculty of Dentistry, Department of Periodontology, Kozlu, Zonguldak 67600, Turkey; 2 Department of Periodontics & Oral Medicine, School of Dentistry, University of Michigan, Ann Arbor, Michigan, USA; 3 Ondokuz Mayıs University, Faculty of Medicine, Department of Pathology, Kurupelit, Samsun, 55210, Turkey

## Abstract

**Introduction:**

Deposition of amyloid fibrils derived from circulating acute-phase reactant serum amyloid A protein causes systemic amyloidosis, a serious inflammatory disorder. We document a male patient who developed reactive amyloidosis (AA type), most likely secondary to his long standing periodontitis.

**Case presentation:**

A 67-year-old Turkish man complained of pain in his oral cavity (burning mouth) especially on the tongue, and had difficulty chewing and swallowing foods. A careful dental/periodontal examination was performed, including assessment of plaque, gingival condition and periodontal probing depths on all his remaining teeth. Prosthetic rehabilitation was provided three months after the completion of his periodontal and surgical therapy. The concentration of serum inflammatory markers including erythrocyte sedimentation rate, white blood cell count, fibrinogen and high sensitive C-reactive protein were measured at baseline, at the second and sixth weeks, and at three and six months after the periodontal and surgical therapy.

**Conclusions:**

Oral examination revealed a few papules on the dorsum of the tongue with two slightly painful, small ulcers, localized on the vestibule of the mouth. The mean probing depth was 9.10 ± 0.84 mm. Biopsies of the tongue, buccal mucosa and retromolar trigone were performed and amyloid deposits were found. The serum inflammatory markers improved more dramatically at the second week of periodontal therapy than any other time intervals.

Amyloidosis may manifest as periodontal destruction that leads to severe chronic periodontitis. Proper periodontal treatment may alleviate systemic inflammatory mediators caused by the amyloidosis.

## Introduction

Reactive systemic AA amyloidosis, with a sustained acute phase response (APR), can complicate chronic inflammatory disorders. AA amyloid fibrils are derived from the acute-phase reactant serum amyloid A protein (SAA) through a process of cleavage, misholding, and aggregation [1]. Renal disease is a frequent manifestation of the systemic amyloidosis and a major cause of morbidity [1]. SAA is an apolipoprotein constituent of high-density lipoprotein that is synthesized by hepatocytes under the transcriptional regulation of pro-inflammatory cytokins [2]. Sustained overproduction of SAA is a prerequisite for the development of AA amyloidosis. Amyloidosis affects a small proportion of patients that present with chronic inflammatory disorders [3,4]. The etiologies for this disease remain unknown. The activation pattern of SAA protein in the presence of inflammation is similar to that of C-reactive protein (CRP) [5]. The level of SAA increases during acute and chronic infections [6,7]. It has been shown that patients with chronic periodontitis display signs of a sub-clinical systemic inflammatory condition [8]. Furthermore, treatment of advanced periodontitis by full-mouth tooth extraction reduced systemic levels of cardiovascular risk and inflammatory reaction [9].

Cross-sectional studies have demonstrated that plasma levels of inflammatory markers such as CRP, fibrinogen, IL-6 and leukocyte counts increase in periodontitis patients when compared to periodontally healthy patients [9,10]. Some studies have shown that effective periodontal therapy reduced levels of CRP [11]. This implies that inflammatory reaction triggered by periodontitis contributes to the whole-body inflammatory burden.

Secondary amyloidosis, representing approximately 45% of all cases of systemic amyloidosis, has been associated with various chronic inflammatory conditions such as rheumatoid arthritis, sarcoidosis, Crohn's disease, ulcerative colitis and tuberculosis [12]. Secondary amyloidosis has also been linked to malignant diseases such as Hodgkin's disease and mesothelioma [12]. In addition, familial Mediterranean fever (FMF), an autosomal recessive disease, primarily affects the population in the Mediterranean basin [13]. FMF is characterized by recurrent episodes of fever and serosal inflammation along with a very intense APR. The most important complication of FMF is secondary amyloidosis [13]. Mutation analysis of Mediterranean fever gene (MEFV) can be helpful in confirming the diagnosis for patients with an atypical presentation. Infection or inflammatory diseases may cause AA amyloidosis even without obvious infection or inflammation [14,15]. The progression of secondary amyloidosis depends on the nature and status of the underlying chronic inflammatory disease. For example, secondary amyloidosis-associated tuberculosis has been shown to undergo remission when the chronic infection has been eliminated [16].

Histopathologic examination of amyloid is essential for the diagnosis and classification of amyloidosis [17,18]. The sensitivity and specificity of the histopathologic diagnosis depend on the biopsy site and the adequacy of the tissue sample [19,20].

## Case presentation

Our patient is 67-year-old Turkish man, a primary school graduate, and a forest ranger who lives in a rustic area. He was fully informed about the study and written consent was obtained from him prior to examination. In his medical examination he explained that his gums started bleeding at a very early age. At age 24, he started to smoke since he thought smoking would help to stop bleeding. Currently, he smokes 1-1.5 packs a day. At age 30, he started to experience difficulty in eating and complained of tooth mobility and gum bleeding. He claimed that his teeth ached a lot and as a result the teeth were extracted by a non-dentist or himself. Consequently, at the age of 50 to 60, he lost most of his teeth. At age 25, he was diagnosed with periodontitis. Nonetheless, he had not seen a dentist for this problem or performed any personal oral hygiene.

Our patient complained of pain in the oral cavity especially on the tongue, buccal mucosa and had difficulty in chewing and swallowing solid food for six months.

**Figure 1:**
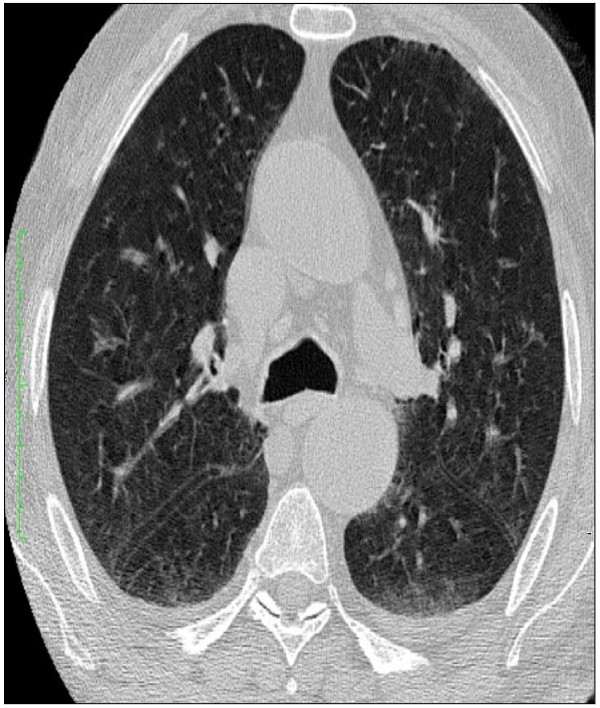
**High resolution computed tomography (HRCT) showed minimal emphysematous areas over both apices and non-specific sequalae**.

His past medical history was significant for a tonsillectomy as a child. All the symptoms in his medical reviews were negative. Spirometric pulmonary tests were normal. High resolution computed tomography (HRCT) showed minimal emphysematous areas over both apices and non-specific sequelae (Figure 1). Head and neck examinations were normal. Magnetic resonance imaging of the tongue revealed no sign of abnormality. He underwent extensive tests to identify the etiologies associated with systemic amyloidosis. Tests such as rectal biopsy, bone marrow biopsy, echocardiogram, abdomen tomography, serum and urine protein electrophoresis as well as liver function test were all normal. However, our patient was diagnosed as chronic renal failure with proteinuria and hypoalbuminemia (Table 1). Renal biopsy showed AA type amyloidosis.

**Table 1:** The effect of periodontal therapy on the inflammatory markers and some laboratory data

Parameters	Before treatment	After treatment			
		**Second week**	**Third week**	**Third month**	**Sixth month**

**ESR (mm/h)**	107	64	60	54	50

**WBC (× 10**^**3 **^**ml)**	18	10.09	9.4	8.5	8.4

**Fibrinogen (mg/dl)**	374.95	280	240	235	236

**hs-CRP(mg/l)**	58	34	30	28	28

**Serum Albumin (g/dl)**	1.8	1.9	2	2.1	2.4

**Serum creatinine (mg/dl)**	3.5	3	3	3	3

**Proteinuria (g/day)**	2.4	1.5	1.5	1.5	1.5

**RF**	Negative				

**MEFV**	Negative				

**FCV**	3.14 (82.4%)				

**FEV**_**1**_	2.67 (105.7%)				

**FEF/FCV**	85%				

**FEF**	2575:2.88(90.6%)				

**HRCT**	Minimal emphysematous areas				

ESR: erythrocyte sedimentation rate (< 10 mm/hour); WBC: white blood cell; Fibrinogen; Serum albumin; hs-CRP: high sensitive C-reactive protein (< 3 mg/L); RF: rheumatoid factor; MEFV: Mediterranean fever gene mutation; FVC: forced vital capacity; FEV1: forced expiratory volume in a second; FEF 25-75: forced expiratory flow 25%-75%; HRCT: high resolution computed tomography

**Figure 2:**
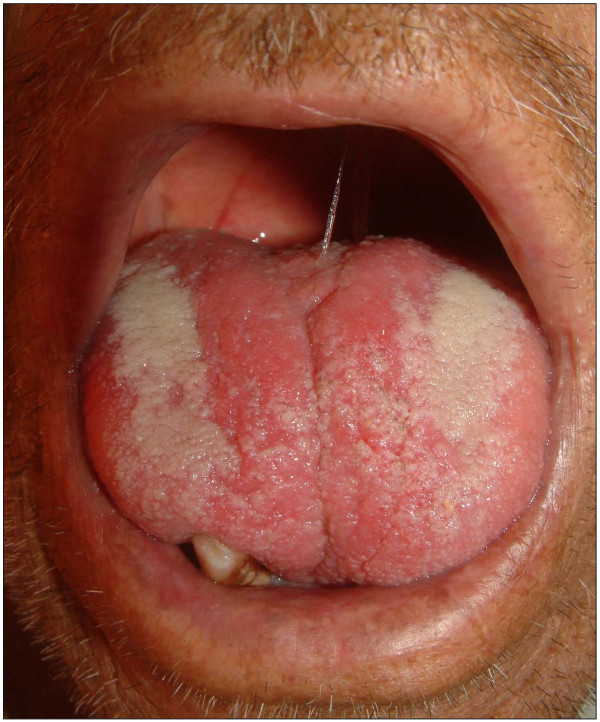
**The tongue was diffusely enlarged (macroglossia), and bilateral white plaques and irregular translucent papules are seen**.

Clinical examination revealed poor oral hygiene and heavy plaque accumulation. Our patient was almost completely edentulous. The mean periodontal probing depth was 9.10 ± 0.84 mm (range 8-10 mm) in his remaining teeth. The tongue was diffusely enlarged (macroglossia) and clear red appearance and bilateral white plaques bleeding easily by gentle removing, and irregular translucent papules were present (Figure 2). Based upon our patient's history, these papules developed spontaneously or after minor trauma. The enlarged tongue has interfered with speech and swallowing, and caused sleep apnea. In addition, two painful, small flat-based ulcers with erythematous halos and a white ulcer bed were detected, localized on the right and left buccal mucosa.

**Figure 3:**
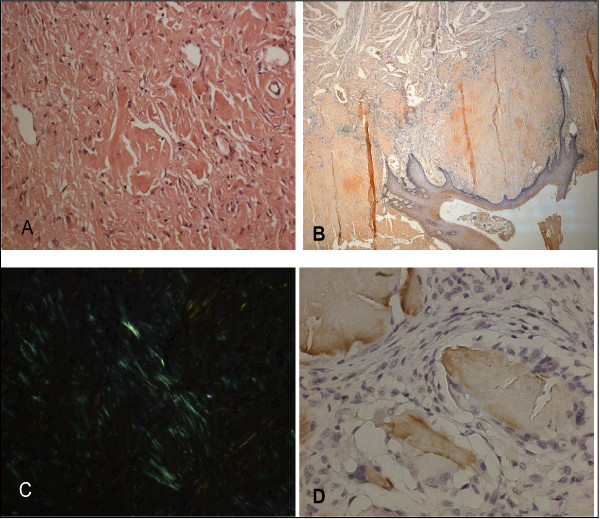
**Histopathologic examination findings: the Congo red method was used to detect amyloid in tissue sections**. Amyloid was identified as the AA type on immunohistochemical testing with the use of monoclonal antibodies specific to SAA. (A) Hematoxylin and eosin staining of the biopsy specimen shows homogen, eosinophilic material (×20, hemotoxylin and eosin). (B) The amorphous extra-cellular material stains positively with Congo red (×2.5, Congo red). (C) This Congo red-positive material appears apple-green when viewed under polarized light (×20 Congo red-positive material appears apple-green when viewed under polarized light). (D) Extra-cellular and peri-vascular deposits of amyloid reveal positive immunoreactivity with an antibody against amyloid A (×40).

Cultures from the plaques on the tongue and oral cavity were all negative for bacteria and fungi. The biopsies were obtained from the tongue, buccal mucosa and retromolar trigon. Secondary amyloidosis (AA type), was diagnosed by histological and immunohistochemical findings (Figure 3).

**Figure 4:**
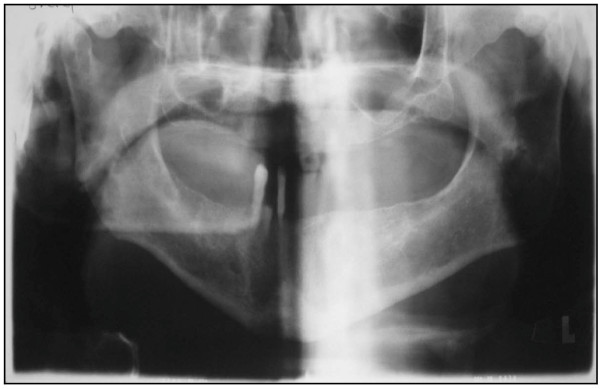
**Radiographic findings from the patient described in this case report**. Diffuse osteopeny in mandibular and prominence trabeculation of maxilla are present.

Our patient received comprehensive periodontal therapy, which included careful oral hygiene instructions, curettage combined with non-surgical and surgical therapy. Serum inflammatory markers and some laboratory data improved dramatically at the second week of the periodontal therapy more than at any other time interval (Table 1). Restorative treatment started after his periodontal condition was stabilized (around three months after periodontal therapy). Intra-oral radiographs showed poor bone density (Figure 4).

### Histopathologic examination findings

The Congo red method was used to detect amyloid in tissue sections [21]. Amyloid was identified as the AA type on immunohistochemical tests with the use of monoclonal antibodies specific to amyloid A [18].

## Discussion

Our patient was 67-year-old man, a heavy and current smoker. He suffered severe chronic periodontitis and chronic renal failure. Our patient complained of pain in the oral cavity especially on the tongue, buccal mucosa and had difficulty in chewing and swallowing solid foods for almost six months. Biopsies were performed and secondary amyloidosis was diagnosed based upon histological and immunohistochemical findings. Our patient presented with typical signs of periodontal disease that include gingival tenderness, bleeding, recession, alveolar bone loss, tooth mobility, and tooth loss. In addition, several inflammation markers, such as erythrocyte sedimentation rate (ESR), white blood cell count (WBC), fibrinogen and high sensitive C-reactive protein (hs-CRP) were all elevated.

It has been shown that chronic infection or inflammatory diseases may cause secondary amyloidosis even without obvious infection or inflammation [14,15]. Patients with chronic periodontal diseases had higher levels of SAA, the precursor protein of amyloid fiber in secondary amyloidosis, than patients without periodontal disease [22]. To date, only a few reports address the interaction between periodontal disease and secondary amyloidosis [20,23]. One study showed the prevalence of moderate to severe periodontitis was greater in FMF patients with amyloidosis than without amyloidosis [20]. The other study was a case report that illustrated an interaction between systemic amyloidosis and severe periodontitis in a patient with rheumatoid arthritis [23]. Our case results were in line with findings reported in the literature.

The definitive method of diagnosing amyloidosis is tissue biopsy. Although biopsies can be obtained from compromised organs, blood vessel fragility associated with amyloid deposition carries a risk of bleeding. Biopsy of oral tissues has been advocated as an adjunctive or alternate method of detecting amyloid deposition. Gingival, tongue, buccal mucosa and minor salivary gland tissue have all been reported as potential sites for biopsy; however, there are inconsistent results with regard to the sensitivity of amyloid detection in each of these tissues [24]. As a result, it has been reported that the anatomic location of the amyloid deposition within the tissue was consistent regardless of the location of the biopsy. This may have important implications for the biopsy technique used for the detection of amyloid [24]. If intra-oral biopsies are used more commonly for patients with chronic periodontal disease, amyloid may be found more frequently than expected.

Our patient's differential diagnosis include pulmonary X-ray, pulmonary function tests, sputum cytology, fasting gastric juice and tuberculin skin testing, they were all negative for tuberculosis and bronchiectasis. His history, physical examination and laboratory findings were negative for rheumatoid arthritis and FMF patients (rheumatoid factor and MEFV gene mutation were negative, respectively). There was no evidence for chronic infection or inflammation such as rheumatoid arthritis, sarcoidosis, Crohn's disease, ulcerative colitis and tuberculosis except chronic periodontitis. In addition, smoking is a strong risk factor for periodontitis [25] and it certainly contributed to our patient's problems since he is a heavy smoker (more than 1-1.5 packs/day).

Secondary amyloidosis is also associated with malignant diseases such as Hodgkin's disease and mesothelioma. Clinical examination, abdominal and chest computed tomography were negative for any malignant disorders or airflow obstruction. With the decline of tuberculosis in the developed countries, rheumatoid arthritis and inflammatory bowel disease remain the leading cause of secondary amyloidosis [12]. However, in the developing countries, chronic infectious diseases such as tuberculosis and leprosy are major causes [12]. There was no clinical evidence for inflammatory bowel diseases or leprosy in our patient.

Although, our patient did not have any pulmonary system complaints, due to his habit of smoking, his pulmonary system was investigated extensively for disease that might trigger the secondary amyloidosis. All pulmonary function tests were normal except a mild emphysematous appearance on chest computed tomography. No bronchiectasis or obstruction was noted. So far, no secondary amyloidosis or increased inflammatory markers in patients with mild or moderate emphysema has been reported [26].

A thorough investigation for the etiology that may cause secondary amyloidosis was carried out. None was identified except chronic periodontitis. Our patient developed reactive amyloidosis (AA-type), most likely secondary to his long-standing periodontitis. This could be attributed to the increase in the levels of systemic inflammatory mediators due to chronic periodontal disease/infection that led to the secondary amyloidosis. Indeed, patients with chronic periodontal diseases have higher levels of SAA protein in secondary amyloidosis than patients without periodontal disease [22]. Chronic periodontal disease could exaggerate secondary amyloidosis via increased levels of systemic inflammatory mediators. In addition, our report highlights the possibility that amyloid deposition in patients with systemic amyloidosis causes accelerated periodontal destruction and bone loss of affected teeth. Amyloid deposition within the periodontium elicited an inflammatory reaction similar to that of foreign body material. Accelerated destruction of periodontium and associated supporting bone apparently is caused by this foreign-body-type giant cell reaction. Therefore, elimination of local infection associated with periodontal diseases will aid in the reduction of levels of systemic inflammatory mediators, which may slow the progression of secondary amyloidosis.

Sustained overproduction of SAA is a prerequisite for the development of AA amyloidosis, although the reasons for these remain unknown. Robbins [27] proposed two possible explanations for this. First, SAA-protein is normally degraded to soluble end products via monocyte-derived enzymes. Conceivably, individuals who develop amyloid have an enzyme defect that cannot breakdown SAA-protein completely hence insoluble AA molecules were produced. Second, a genetically determined structural abnormality in the SAA-protein molecule itself renders it resistant to degradation by monocytes. Evidence has suggested that individual genetic susceptibility to amyloidosis may influence the host's response to infection. Nibali *et al*. [28] have found the link between polymorphisms of genes encoding for neutrofil receptors and pro-inflammatory cytokines and the presence of pathogenic bacteria in patients with aggressive periodontits. The authors then speculated that complex interactions between the microbiota and host genome may be at the basis of a patient's susceptibility to aggressive periodontitis. Currently many investigators are trying to define the genotype-phenotype correlations and risk factors for the development of secondary amyloidosis.

## Conclusions

To our knowledge, this is the first case report that documents secondary amyloidosis supported by the tongue, buccal mucosa and retromolar trigon biopsies, while ruling out all possible known etiologic factors as a cause for secondary amyloidosis. In addition, this study has demonstrated that secondary amyloidosis can be slowed down if periodontal condition can be improved. Further studies in a larger population will provide insight of this rare but destructive systemic disease.

## Abbreviations

APR: acute-phase reactant; CRP: C-reactive protein; ESR: erythrocyte sedimentation rate; FMF: familial Mediterranean fever; hs-CRP: fibrinogen and high sensitive C-reactive protein; MEFV: mutation analysis of Mediterranean fever gene; SAA: serum amyloid A protein; WBC: white blood cell count.

## Consent

Written informed consent was obtained from the patient for publication of this case report and any accompanying images. A copy of the written consent is available for review by the Editor-in-Chief of this journal.

## Competing interests

The authors declare that they have no competing interests.

## Authors' contributions

MİC and HW analyzed and interpreted our patient data. They were also major contributors in writing the manuscript. LY performed the histological examination of the tissue biopsies. All the authors read and approved the final manuscript
